# Identification of Genes Influencing Skeletal Phenotypes in Congenic P/NP Rats

**DOI:** 10.1002/jbmr.8

**Published:** 2010-01-29

**Authors:** Imranul Alam, Lucinda G Carr, Tiebing Liang, Yunlong Liu, Howard J Edenberg, Michael J Econs, Charles H Turner

**Affiliations:** 1 Departments of Biomedical Engineering, Indiana University Purdue University Indianapolis (IUPUI)Indianapolis, IN, USA; 2 Medicine, Indiana University Purdue University Indianapolis (IUPUI)Indianapolis, IN, USA; 3 Pharmacology, Indiana University Purdue University Indianapolis (IUPUI)Indianapolis, IN, USA; 4 Biochemistry and Molecular Biology, Indiana University Purdue University Indianapolis (IUPUI)Indianapolis, IN, USA; 5 Biomechanics and Biomaterials Research Center, Indiana University Purdue University Indianapolis (IUPUI)Indianapolis, IN, USA

**Keywords:** bone mass, congenic, QTL, neuropeptide Y, gene

## Abstract

We previously showed that alcohol-preferring (P) rats have higher bone density than alcohol-nonpreferring (NP) rats. Genetic mapping in P and NP rats identified a major quantitative trait locus (QTL) between 4q22 and 4q34 for alcohol preference. At the same location, several QTLs linked to bone density and structure were detected in Fischer 344 (F344) and Lewis (LEW) rats, suggesting that bone mass and strength genes might cosegregate with genes that regulate alcohol preference. The aim of this study was to identify the genes segregating for skeletal phenotypes in congenic P and NP rats. Transfer of the NP chromosome 4 QTL into the P background (P.NP) significantly decreased areal bone mineral density (aBMD) and volumetric bone mineral density (vBMD) at several skeletal sites, whereas transfer of the P chromosome 4 QTL into the NP background (NP.P) significantly increased bone mineral content (BMC) and aBMD in the same skeletal sites. Microarray analysis from the femurs using Affymetrix Rat Genome arrays revealed 53 genes that were differentially expressed among the rat strains with a false discovery rate (FDR) of less than 10%. Nine candidate genes were found to be strongly correlated (*r*^2^ > 0.50) with bone mass at multiple skeletal sites. The top three candidate genes, *neuropeptide Y* (*Npy*), *α synuclein* (*Snca*), and *sepiapterin reductase* (*Spr*), were confirmed using real-time quantitative PCR (qPCR). Ingenuity pathway analysis revealed relationships among the candidate genes related to bone metabolism involving β-estradiol, interferon-γ, and a voltage-gated calcium channel. We identified several candidate genes, including some novel genes on chromosome 4 segregating for skeletal phenotypes in reciprocal congenic P and NP rats. © 2010 American Society for Bone and Mineral Research.

## Introduction

Osteoporosis is a common multifactorial disorder characterized by reduced bone mass and microarchitectural deterioration of bone tissue, leading to reduced bone strength and increased susceptibility to fracture.(1) Bone mineral density (BMD), the most important surrogate for osteoporotic fracture, is strongly heritable at every skeletal site.(2,3) As much as 80% of peak BMD and about a third of the variance in the risk of fracture have been found to be attributable to genetic factors.(3) Although linkage studies in human and animal models have identified many quantitative trait loci (QTLs) for different bone phenotypes,(4,5) the causal genes underlying these phenotypes have yet to be discovered.

In a previous study we compared bone phenotypes in inbred alcohol-preferring (P) and alcohol-nonpreferring (NP) rats.(6) These rat lines were developed at Indiana University for high and low alcohol preference behavior by selective breeding from a randomly bred closed colony of Wistar rats [Wrm: WRC (WI)BR from Walter Reed Army Institute of Research, Washington, DC].(7) After selective breeding for 30 generations, inbreeding was initiated and continued for another 20 generations to obtain inbred P and NP lines. Using these inbred lines, we demonstrated that P rats have significantly higher BMD than NP rats both in long bones and in lumbar vertebrae.(6) Furthermore, P rats have larger cross-sectional area and stronger long bones than NP rats. Using genome-wide linkage analysis, we identified several QTLs on chromosomes 4, 5, 10, 12, and 16 influencing alcohol preference in P and NP rats.(8) The major QTL for alcohol preference was observed in the region between q22 and q34 on chromosome 4, with an logarithms of odds (LOD) score of 9.2. Interestingly, in a separate linkage study using inbred Fischer 344 (F344) and Lewis (LEW) rats, several QTLs linked to bone density and structure were identified at the same location,(9,10) suggesting that some novel bone mass–regulating genes might have segregated during selective breeding for the alcohol-preference trait.

Identification of candidate genes following the discovery of QTLs for a complex disease such as osteoporosis requires multiple approaches because the linkage region is usually broad and harbors hundreds of genes. The development of a congenic animal model through a series of backcrossings is the first step to confirm a QTL and narrow down the QTL interval. By exploiting this approach, we have created reciprocal congenic rats (P.NP and NP.P) by introgressing the 4q22-q34 QTL region of one inbred strain (donor) into the genetic background of another inbred strain (recipient).(11) In order to identify the effects of transfer of the QTL on skeletal phenotypes, we measured multiple bone phenotypes, including total BMD, bone mineral content (BMC), total bone area, and biomechanical properties at different skeletal sites in inbred and congenic P and NP rats. Since body weight and activities might influence the bone phenotypes, we compared the bone phenotypes in weight-bearing (femur) versus non-weight-bearing (cranium) sites and measured daily activity levels in these rats.

The purpose of this study is to identify genes segregating for bone phenotypes in congenic P and NP rats. We performed microarray-based gene expression analysis to identify the candidate genes underlying the variations in skeletal phenotypes in inbred and congenic P and NP rats. The differentially expressed genes were ranked based on the proportion of the variation in skeletal phenotypes explained by the expression level of each gene. In addition, we used a network-based pathway analysis tool to identify the known functional interrelationships among these candidate genes.

## Materials and Methods

### Animals

We used 16 inbred male P and NP rats and 16 congenic male rats (*n* = 8 per strain) derived from inbred P and NP rats. Generation of each reciprocal congenic rat line (P.NP and NP.P) involved transfer of the 4q22-q34 QTL region (demarcated by the flanking markers D4Mgh16 at 34 cM and D4Rat55 at 55.5 cM) from one inbred strain (donor) into the genetic background of another inbred strain (recipient), as described previously.(11) All rats were developed and maintained at Indiana University. Transfer of the donor region was accomplished by first producing (P × NP or NP × P) N_1_F_1_ offspring and then backcrossing an N_1_F_1_ rat to a recipient rat to obtain N_2_F_1_ progeny. Ten generations of backcrossing were performed, followed by an intercross between N_10_ animals to produce homozygous N_10_F_1_ animals, which resulted in the congenic strains. The nomenclature for congenic strains lists the recipient strain first and the donor strain second. Therefore, NP.P has the QTL at 4q22-4q34 donated from the P onto the NP background. Rats were individually housed in polycarbonate cages in a vivarium maintained on a 12-hour light/dark cycle on sterilized northern white pine shavings bedding and provided standard rat chow (NIH-31 Mouse/Rat Diet 7017, Harlan Teklad, Madison, WI, USA) and water *ad libitum*. The procedures performed throughout the experiment followed the guidelines of the Indiana University Animal Care and Use Committee (IACUC).

### Euthanasia and specimen collection

Rats were euthanized by cervical dislocation at 6 months of age, and lower limbs and lumbar vertebrae (L_1_–L_6_) were dissected out. The lower limbs on the right side were immediately stored at –20°C for biomechanical testing. The lower limbs on the left side were stripped of the muscle and transferred to 70% ethyl alcohol and stored at 4°C for densitometry analyses.

### DNA isolation and genotyping

Isolation of genomic DNA and genotyping of each rat were accomplished as described previously.(11)

### Cage activity test

Rats were assessed for motor activity for 1 hour in a Digiscan animal activity monitor (Model VMRXYZ TAO, AccuScan Instruments, Columbus, OH, USA) with dimensions of 42 × 42 × 30 cm during both light and dark cycles. There were 16 beams to detect horizontal or vertical movement. Beam spacing for all sensors was 2.5 cm. All walls of the activity chambers were composed of clear acrylic sheet. Activity chambers were connected to the VersaMax/Digiscan analyzer (Model CDA-8, AccuScan Instruments) for relay of movement data to the Digipro software system (Versadat Version 1.50, AccuScan Instruments). Activity chambers were cleaned thoroughly between tests. The average activity was determined from both horizontal and vertical movements during light and dark cycles.

### Dual energy X-ray absorptiometry (DXA)

Whole-body and whole-cranial BMC and areal BMD (aBMD) were measured using a fan-beam Hologic QDR 4500A DXA machine (Hologic, Bedford, MA, USA) equipped with Hologic Version 11.2:3 software and a 1.698-mm-diameter collimator, with line spacing of 0.0311 cm, point resolution of 0.0311 cm, and acquisition time of 149 seconds. The machine was calibrated daily with an anthropomorphic spine phantom. After completion of the scan, mutually exclusive region-of-interest (ROI) boxes were drawn around the whole body and the cranium from which aBMD and BMC were obtained. BMC was normalized by body weight (BW) to adjust for differences in body size among the rat lines.

The left femur and lumbar vertebrae 1 to 6 (L_1_–L_6_) were scanned using DXA (PIXImus II Mouse Densitometer, Lunar Corp., Madison, WI, USA) with ultrahigh resolution (0.18 × 0.18 mm/pixel). During scanning, dissected femurs were positioned with the lateral surface of the diaphysis facing down on a platform supplied by the manufacturer. After completion of the scan of each bone, mutually exclusive ROI boxes were drawn around the bone from which BMC/BW measurements were obtained.

### Peripheral quantitative computed tomography (pQCT)

The left femur, proximal ends of the left femurs, and the fifth lumbar vertebra (L_5_) were placed in plastic tubes filled with 70% ethyl alcohol and centered in the gantry of a Norland Stratec XCT Research SA+ pQCT System (Stratec Electronics, Pforzheim, Germany). Two cross-sectional levels were scanned for femur—one at the midshaft and one at the distal femur. The distal slices were scanned approximately 1 mm below the growth plate. For the femoral neck, five consecutive scans perpendicular to the neck axis were obtained 0.25 mm apart starting at the base of the femoral head and ending at the greater trochanter. The lumbar vertebrae were scanned for a single slice through the caudocranial center of the vertebral body. A single tomography slice of 0.26-mm thickness was taken at the collimation of 4 × 10^5^ counts/s and at a voxel size of 0.07 mm. For each slice, the X-ray source was rotated through 180 degrees of projection for one block. The slice through the femoral midshaft and neck included mainly cortical bone, whereas the slices from distal femur and lumbar vertebra included both cortical and trabecular bones. For each slice of femoral midshaft, distal femur, and L_5_, total volumetric BMD (vBMD) was obtained using XCT Research SA+ Software Version 5.40 (Stratec Electronics). For the femoral neck, total vBMD was measured from the average values of all five slices pQCT images. Density thresholds of 500 and 900 mg/cm^3^ were used to identify mineralized bone.

### Biomechanical testing

The frozen right femurs were brought to room temperature slowly in a saline bath. The femurs were tested in three-point bending by positioning them on the lower supports of a three-point bending fixture and applying load at the midpoint using a material testing machine (Alliance RT/5, MTS Systems Corp., Eden Prairie, MN, USA). The bones were held in place by a small (1-N) preload and then loaded in monotonic axial compression until fracture at a crosshead speed of 20 mm/min. Load was applied midway between two supports that were 20 mm apart. After the long bones were fractured, cortical thickness was measured at the midshaft and 5 mm distal and proximal to the midshaft using digital calipers accurate to 0.01 mm and with a precision of +0.005 mm (Mitutoyo, Aurora, IL, USA). For femoral neck, the proximal half of each femur was mounted vertically in a special chuck that clamped the femoral shaft to the lower platen of the same materials testing machine. Load was applied downward onto the femoral head at a crosshead speed of 20 mm/min until the femoral neck fractured. Force and displacement measurements were collected every 0.05 second. From the force versus displacement curves, ultimate force (*F*_*u*_, in N) was calculated in TestWorks Software Version 4.06 (Eden Prairie, MN).

### RNA extraction and microarray measurements

Femurs from 4-week-old P, NP, NP.P, and P.NP animals were harvested and immediately frozen in liquid nitrogen and stored at −80°C until required. RNA from the femurs was extracted (*n* = 5 per strain) using Trizol (Invitrogen, Carlsbad, CA, USA), followed by further purification using an RNeasy Mini Kit (Qiagen, Inc., Valencia, CA, USA) according to manufacturer's instructions. RNA then was treated with a DNA-free kit (Ambion, Austin, TX, USA) to remove any residual genomic DNA. The quality of RNA was determined using a 2100 Bioanalyzer (Agilent Technologies, Palo Alto, CA, USA) and was quantified using a spectrophotometer (NanoDrop, Wilmington, DE, USA). For microarray analysis, 5 µg of total RNA from each sample was processed according to the standard protocols from Affymetrix (*GeneChip Expression Analysis Technical Manual*, Affymetrix, Santa Clara, CA, USA), and 10 µg of cRNA from each sample was hybridized to a separate Rat Genome 230 2.0 Array (P/N 511056, Affymetrix) for 17 hours at 45°C with constant rotation. The GeneChip then was washed and stained in the Affymetrix Fluidics Station 400 according to the standard protocol. Subsequently, each array was scanned by the Agilent GeneChip Scanner 3000 (Agilent Technologies). All procedures were carried out using a balanced design. The Rat Genome 230 Array has 31,000 probe sets representing 28,700 well-substantiated rat genes.

### Quality control (QC) for RNA and Affymetrix data

Measurement of the ratio between signals from the 5′ and 3′ ends of the *GAPDH* and *β-actin* genes (3′/5′ ratios) and the RNA degradation plots were used for determination of RNA quality. Affymetrix data QC was done by determining the percentage of present or detection calls and the scale factors between the arrays. To ensure that the overall gene expression profiles in all the samples in each experimental condition were correctly correlated, principal-component analysis was conducted.

### Microarray data analysis and informatics

The images from each array were analyzed using an Affymetrix GeneChip Operating System (GCOS) with Version 1.2 software. The .cel files were analyzed in the statistical programming environment R(12) with tools available from the Bioconductor Project.(13) The normalization and log_2_ transformation of all expression data were done using the robust multichip average (RMA) method(14,15) implemented in the Bioconductor RMA. Affymetrix data were used for mapping of all probe sets to their chromosomal location. The identities of the probe sets were confirmed by comparing the target mRNA sequences on the Affymetrix Rat Genome 230 2.0 GeneChip with the National Center for Biotechnology Information (NCBI) GenBank database (http://www.ncbi.nlm.nih.gov/Genbank/). Only probe sets that were reliably detected (called *present* by the detection call generated by the Affymetrix Microarray Analysis Suite 5.0 algorithm) were analyzed; this reduces false positives.(16)

### Culture of primary osteoblasts

Calvaria were harvested from newborn P, NP, NP.P, and P.NP pups (*n* = 5 to 8 per strain); cleaned of all loosely adherent fibrous tissue, periosteum, and dura mater; and minced. The dissected calvaria then were digested sequentially for seven times each for 15 minutes with 0.5 mg/mL of crude collagenase P (Roche Molecular Biochemical, Indianapolis, IN, USA) in a solution of 3 mL of trypsin/EDTA at room temperature with gentle rocking. The supernatant from the first digestion was discarded, and from each subsequent digest (digests 2 to 7), released cells were collected. The pooled solution from digests 2 to 7 then was filtered through a Nitex membrane (Millipore Corp., Bedford, MA, USA), centrifuged, and resuspended in α minimum essential medium (α-MEM) (Invitrogen). Cells were placed in 75-cm^2^ flasks and grown in α-MEM supplemented with 10% fetal bovine serum (FBS) and antibiotics (100 IU/mL of penicillin, 100 µg/mL of streptomycin) at 37°C in a humidified atmosphere of 5% CO_2._ Once cells reached about 80% confluence, they were resuspended with 0.05% trypsin in EDTA and plated onto 75-cm^2^ flasks. First-passage primary osteoblasts were used for subsequent RNA isolation.

### Real-time PCR measurements

Top candidate genes from microarray data were verified using real-time quantitative PCR (qPCR). Two micrograms of total RNA (the same RNA used for Affymetrix analysis; *n* = 3) was reverse transcribed using Superscript III reverse-transcription reagent for first-strand cDNA synthesis (Invitrogen). All PCR reactions contained the first-strand cDNA corresponding to 100 ng of total RNA. TaqMan predeveloped primers, FAM-dye-labeled MGB probes, and universal mastermix (Applied Biosystems, Foster City, CA, USA) were used to quantity the relative gene expression. Rat *GAPDH* was used as an endogenous control. Real-time detection of PCR products was performed using an ABI PRISM 7300 sequence detector (Applied Biosystems). Relative expression of mRNA was calculated based on a relative standard curve and normalized to *GAPDH*. All real-time PCR analyses used triplicates of each of three biologic samples.

### Statistics

To detect significant differences for bone phenotypes among all rat strains, one-way ANOVA analysis was performed, followed by Fischer's protected least-significance differences. The level of significance was set at .05. For BW correction of BMC and polar moment of inertia (Ip), rat strains were compared using ANOVA with BW as a covariate. For microarray analysis, *p* values among all strains were calculated by ANOVA using the package Limma.(17) For comparison of differentially expressed *trans-*regulated genes, we used the Welch *t* test between the strains (NP versus NP.P and P versus P.NP). Because our hypothesis was that *cis*-regulated genes within the introgressed regions would be differentially regulated, the 460 probe sets that mapped to the introgressed chromosome 4 QTL region (and therefore were potentially *cis*-regulated) were analyzed separately. False discovery rate (FDR) was calculated by the method of Benjamini and Hochberg.(18) Probe sets were considered differentially expressed if the FDR was less than 10%. The microarray data set was submitted to the NCBI Gene Expression Omnibus (GEO) Express Web portal (GEO Accession Number GSE 12066). For each phenotype of interest (ie, whole-body, cranial, femur, and L_1_–L_6_ BMC/BW; whole-body and cranial aBMD; femur midshaft, distal femur, and L_5_ total vBMD; and femur and femoral neck ultimate force), regression analysis was performed with the average gene expression level for the strain as the dependent variable and the phenotypic mean value in animals of that strain as the independent variable. The proportion of variation (*r*^2^ value) in the phenotypic means explained by the variation in gene expression was obtained using the statistical software package StatView (Abacus Concepts, Inc., Berkeley, CA, USA).

### Pathway analysis

The interactions among differentially expressed genes for each bone phenotype were investigated using Ingenuity Pathway Analysis (IPA 5.0, Ingenuity Systems, Inc., Mountain View, CA, USA). Differentially expressed genes that explained a significant proportion of the variation in bone phenotypes were uploaded into the application. Each gene identifier was mapped to its corresponding gene in the Ingenuity Pathway Knowledge Base (IPKB). These genes were overlaid onto a global network developed from the information contained in the IPKB. Networks of these genes, defined as the reflection of all interactions of a given gene defined in the literature, then were generated algorithmically based on their connectivity. The interactions indicate physical association, induction/activation, or repression/inactivation of one gene product by the other, directly or through another intermediary molecule.

## Results

### Effect of QTL transfer on body weight and cage activity

P rats had lower BWs and higher activity levels than NP rats (Table 1). NP.P rats, containing the P 4q22-q34 QTL on the NP background, had significantly lower BWs and increased activity compared with NP rats. P.NP rats, with the NP QTL on the P background, had significantly higher BWs but did not differ significantly from the background P strain in activity level.

**Table 1:** Body weight and cage activities in NP, NP.P, P.NP and P rats (*n* = 8)^a^

	Strains				ANOVA p-value			
		
Phenotypes	NP	NP.P	P.NP	P	NP/P	NP/NP.P	P/P.NP	NP.P/P.NP
Body Weight (g)	639 ± 29	609 ± 33	597 ± 24	543 ± 29	<0.0001	0.01	0.0002	0.47
Average activity	5126 ± 1722	7290 ± 2435	7849 ± 1484	8194 ± 972	0.001	0.02	0.7	0.54

a Values are mean ± standard deviation (SD).

### Effect of QTL transfer on bone mass

#### Whole-body and cranial aBMD and BMC measured with DXA

P rats had significantly higher whole-body and cranial aBMD and BMC/BW than NP rats (Table 2). NP.P rats had higher whole-body and cranial aBMD and whole-body BMC/BW than NP rats, demonstrating that the presence of the P QTL increased BMD in NP rats. Conversely, P.NP rats had significantly lower whole-body and cranial aBMD and BMC/BW than P rats, indicating that the presence of the NP QTL lowered BMD.

**Table 2:** Bone Phenotypes for whole body, cranium, femur, femoral neck and lumbar vertebrae in NP, NP.P, P.NP and P rats (*n* = 8)^a^

	Strains				ANOVA *p*-value			
		
Phenotypes	NP	NP.P	P.NP	P	NP/P	NP/NP.P	P/P.NP	NP.P/P.NP
DXA								
Whole body aBMD (g/cm^2^)	0.188 ± 0.003	0.200 ± 0.003	0.203 ± 0.005	0.210 ± 0.005	<0.0001	<0.0001	0.002	0.19
Whole body BMC/BW	0.02184 ± 0.0003	0.02276 ± 0.001	0.02330 ± 0.001	0.02392 ± 0.001	<0.0001	0.007	0.05	0.09
Cranial aBMD (g/cm^2^)	0.371 ± 0.01	0.389 ± 0.01	0.389 ± 0.01	0.416 ± 0.01	<0.0001	0.01	0.0003	0.9
Cranium BMC/BW	0.0062 ± 0.0002	0.0065 ± 0.0003	0.0064 ± 0.0002	0.0067 ± 0.0004	0.001	0.08	0.01	0.28
Femur aBMD (g/cm^2^)	0.244 ± 0.004	0.232 ± 0.005	0.235 ± 0.01	0.246 ± 0.01	0.6	0.01	0.02	0.54
Femur BMC/BW	0.00114 ± 0.0001	0.00123 ± 0.0001	0.00129 ± 0.0001	0.00133 ± 0.0001	<0.0001	0.006	0.11	0.03
Lumbar 1-6 aBMD (g/cm^2^)	0.218 ± 0.01	0.223 ± 0.01	0.236 ± 0.01	0.238 ± 0.01	<0.0001	0.27	0.75	0.004
L1-6 BMC/BW	0.00220 ± 0.0001	0.00235 ± 0.0001	0.00249 ± 0.0001	0.00258 ± 0.0001	<0.0001	0.006	0.01	0.001
pQCT								
Femur midshaft total vBMD (mg/cm^3^)	952 ± 11	966 ± 25	960 ± 14	1044 ± 21	<0.0001	0.18	<0.0001	0.53
Distal femur total vBMD (mg/cm^3^)	588 ± 19	582 ± 24	618 ± 17	653 ± 24	<0.0001	0.64	0.004	0.005
Femoral neck total vBMD (mg/cm^3^)	1117 ± 17	1092 ± 39	1033 ± 33	1082 ± 35	0.04	0.16	0.007	0.002
Lumbar 5 total vBMD (mg/cm^3^)	584 ± 24	571 ± 19	626 ± 18	685 ± 31	<0.0001	0.3	<0.0001	0.0002
Femur Ip/BW (mm^4^/g)	0.00816 ± 0.001	0.00851 ± 0.0003	0.00840 ± 0.0002	0.00824 ± 0.0002	0.71	0.04	0.43	0.64
Femoral neck Ip/BW (mm^4^/g)	0.00057 ± 0.0001	0.00069 ± 0.0001	0.00125 ± 0.0001	0.00132 ± 0.0001	<0.0001	0.11	0.36	<0.0001
Femur ultimate force (N)	142 ± 13	157 ± 16	153 ± 5	159 ± 13	0.01	0.03	0.41	0.61
Femoral neck ultimate Force (N)	143 ± 9	134 ± 12	155 ± 15	174 ± 17	0.0003	0.27	0.01	0.01

a Values are mean ± standard deviation (SD).

It is noteworthy that we detected higher BMD in P rats than in NP rats at a non-weight-bearing area such as the cranium, and the presence of the NP QTL significantly reduced bone mass in the cranium, suggesting that the skeletal phenotypic differences in these rats could not be explained only by increasing physical activity levels.

#### Whole-femur and L_1_–L_6_ aBMD and BMC measured with DXA

P rats had a significantly higher whole-femur BMC/BW and L_1_–L_6_ aBMD and BMC/BW than NP rats (Table 2). NP.P rats had a higher femur BMC/BW and L_1_–L_6_ BMC/BW than NP rats, indicating that the presence of the P QTL increased bone mass. Conversely, the presence of the NP QTL lowered bone mass because P.NP rats had significantly lower femur aBMD and L_1_–L_6_ BMC/BW than P rats.

#### Femur midshaft, distal femur, femoral neck, and L_5_ total vBMD measured with pQCT

P rats had a significantly higher total vBMD at most skeletal sites (i.e., femur midshaft, distal femur, and L_5_) compared with NP rats (Table 2). An exception was found at the femoral neck, where total vBMD was significantly lower in P rats than in NP rats. There were no differences in total vBMD for any of these sites between NP and NP.P rats, suggesting that the presence of the P QTL in the NP background did not have much effect on vBMD. However, the presence of the NP QTL in the P background significantly lowered total vBMD at all these sites.

### Effect of QTL transfer on Ip and bone strength

P rats had significantly more robust and stronger bones than NP rats, as evidenced by significantly higher femoral neck Ip/Body Weight (BW) and significantly greater ultimate force at the femur and femoral neck (UF) (Table 2). NP.P rats had a significantly higher femur Ip/BW and UF than NP rats, demonstrating that the P QTL improved bone structure and strength. P.NP rats had a significantly lower femoral neck UF than P rats, indicating that the transfer of the NP QTL lowered bone strength. However, no significant differences for femur and femoral neck Ip/BW were observed between P and P.NP rats.

### Effect of QTL transfer on gene expression

#### Microarray analyses

The Affymetrix microarray analyses using RNA from femurs showed that a total of 53 genes, residing in the chromosome 4 QTL region, including 41 candidate genes and 12 predicted genes, were differentially expressed (FDR <0.10) among the rat strains (Table 3). We used the term *candidate gene* to refer to the differentially expressed known genes with a setting of FDR <0.10. In addition, *predicted genes* are the genes indicated as “predicted” in NCBI GenBank Database with the same setting. Regression analyses were performed to assess the correlation between gene expression and skeletal phenotypes. Genes with a strong correlation (*r*^2^ > 0.50) in at least one phenotype of interest are indicated in bold in Table 3. These were prioritized based on the strength of correlation for average BMC derived from whole-body, cranium, and hind limb BMC. A total of nine candidate genes were found to be strongly correlated with average BMC (Table 3).

**Table 3:** R-square values and fold-change for differentially expressed cis-regulated genes by microarray analysis within 4q22-q34 region on chromosome 4 in NP, NP.P, P.NP and P rats^a^^b^

		R-square values												Fold change						
						
Gene symbol	Gene name	Average BMC	Whole body BMC/BW	Cranial BMC/BW	Femur BMC/BW	L1-6 BMC/BW	Whole body aBMD	Cranial aBMD	Femur Midshaft Total vBMD	Distal Femur Total vBMD	L5 Total vBMD	Femur Ultimate Force	Femur Neck Ultimate Force	NP vs P	NP vs NP.P	P vs P.NP	NP.P vs P.NP	ANOVA p-value	FDR-Chr 4^c^	FDR-Chr all^d^
Candidate genes	Candidate genes																			
Snca	Synuclein, alpha	**0.89**	**0.90**	**0.98**	**0.85**	**0.83**	**0.95**	**0.96**	**0.73**	**0.59**	**0.56**	**0.88**	0.46	1.7	1.3	0.8	1.0	0	0	0.00006
Spr	Sepiapterin reductase	**0.86**	**0.86**	**0.97**	**0.83**	**0.79**	**0.94**	**0.89**	**0.62**	0.47	0.44	**0.95**	0.34	1.5	1.3	0.8	0.9	0.0007	0.008	0.009
Npy	Neuropeptide Y	**0.73**	**0.73**	**0.76**	**0.75**	**0.67**	**0.82**	**0.62**	0.28	0.23	0.20	**0.97**	0.13	1.5	1.4	0.9	0.9	0.00001	0.0009	0.0009
Arf5	ADP-ribosylation factor 5	**0.65**	**0.63**	**0.89**	**0.54**	**0.55**	**0.67**	**0.91**	**0.95**	**0.58**	**0.59**	**0.59**	**0.51**	0.8	0.9	1.2	1.1	0.00001	0.0003	0.07
Tmem209	Transmembrane protein 209	**0.64**	**0.72**	0.28	**0.77**	**0.81**	**0.61**	0.44	0.26	**0.72**	**0.68**	0.27	**0.66**	1.2	1.0	1.0	1.2	0.002	0.02	0.02
Slc25a26	Solute carrier family 25 (mitochondrial carrier, phosphate carrier), member 26	**0.60**	**0.68**	0.26	**0.70**	**0.76**	**0.55**	0.45	0.33	**0.82**	**0.79**	0.20	**0.79**	1.1	1.0	1.0	1.2	0.0004	0.008	0.09
Gpnmb	Glycoprotein (transmembrane) nmb	**0.56**	**0.50**	**0.88**	0.45	0.40	**0.62**	**0.69**	**0.53**	0.19	0.17	**0.84**	0.11	0.7	0.7	1.3	1.2	0.006	0.07	0.06
Fkbp14	FK506 binding protein 14	**0.52**	**0.58**	0.17	**0.66**	**0.67**	**0.50**	0.26	0.07	0.43	0.39	0.26	0.36	0.9	0.9	0.9	0.8	0.002	0.03	0.03
Isy1	ISY1 splicing factor homolog	**0.51**	**0.58**	0.16	**0.62**	**0.67**	0.46	0.32	0.20	**0.69**	**0.66**	0.14	**0.66**	1.2	1.0	1.0	1.2	0.002	0.02	0.02
Ghrhr	Growth hormone releasing hormone receptor	0.48	0.42	0.84	0.35	0.32	**0.51**	**0.71**	**0.69**	0.24	0.24	**0.65**	0.17	0.6	0.7	1.6	1.4	0.003	0.04	0.04
Nup205	Nucleoporin 205	0.47	0.53	0.13	**0.57**	**0.63**	0.41	0.28	0.17	**0.66**	**0.63**	0.11	**0.64**	1.1	1.0	1.0	1.2	0.004	0.03	0.03
Skap2	Src family associated phosphoprotein 2	0.46	0.43	0.64	0.43	0.35	**0.55**	0.42	0.17	0.05	0.03	**0.89**	0.01	1.3	1.5	0.9	0.8	0.00004	0.0003	0.0005
Ruvbl1	RuvB-like protein 1	0.45	0.51	0.12	**0.56**	**0.61**	0.39	0.26	0.14	**0.63**	**0.60**	0.10	**0.61**	1.1	1.0	1.1	1.2	0.002	0.02	0.02
Tmem43	Transmembrane protein 43	0.44	0.50	0.10	**0.57**	**0.60**	0.41	0.20	0.06	0.45	0.41	0.16	0.40	0.9	1.0	0.9	0.8	0.006	0.05	0.05
Wdr91	WD repeat domain 91	0.44	0.37	0.80	0.30	0.28	0.46	**0.64**	**0.62**	0.18	0.17	**0.64**	0.12	1.4	1.3	0.7	0.8	0.0006	0.007	0.008
Tmem176b	Transmembrane protein 176B or Torid	0.38	0.33	0.60	0.33	0.26	0.46	0.36	0.15	0.02	0.01	**0.82**	0.00	1.3	1.4	0.9	0.8	0.0004	0.006	0.006
Sec61a1	Sec61 alpha 1 subunit	0.36	0.41	0.06	0.48	**0.51**	0.31	0.14	0.04	0.42	0.39	0.09	0.40	1.1	1.0	1.1	1.3	0.003	0.03	0.03
Bpgm	2,3-bisphosphoglycerate mutase	0.36	0.42	0.09	0.43	**0.51**	0.30	0.26	0.23	**0.73**	**0.73**	0.04	**0.77**	1.2	0.9	1.0	1.4	0.0003	0.01	0.01
Cald1	Caldesmon 1	0.32	0.37	0.07	0.38	0.45	0.25	0.22	0.21	**0.71**	**0.70**	0.02	**0.75**	0.9	1.1	1.0	0.8	0.003	0.03	0.03
Abtb1	Ankyrin repeat and BTB (POZ) domain containing 1	0.27	0.31	0.02	0.34	0.40	0.20	0.13	0.09	**0.55**	**0.53**	0.01	**0.58**	1.1	0.9	1.1	1.3	0.001	0.01	0.01
Add2	Adducin 2 (beta)	0.26	0.30	0.06	0.29	0.37	0.19	0.22	0.27	**0.72**	**0.73**	0.00	**0.80**	1.2	0.8	1.0	1.3	0.0004	0.008	0.009
Gars	Glycyl-tRNA synthetase	0.22	0.25	0.10	0.22	0.29	0.16	0.28	0.46	**0.75**	**0.79**	0.00	**0.86**	1.1	0.9	0.9	1.1	0.01	0.09	0.08
Gadd45a	Growth arrest and DNA-damage-inducible 45 alpha	0.20	0.24	0.01	0.25	0.32	0.14	0.11	0.11	**0.56**	**0.56**	0.00	**0.62**	1.1	0.8	1.1	1.5	0.00005	0.001	0.001
Cnbp	Cellular nucleic acid binding protein	0.19	0.22	0.02	0.22	0.29	0.12	0.15	0.21	**0.64**	**0.65**	0.00	**0.73**	1.1	0.9	1.0	1.1	0.008	0.06	0.06
Chn2	Chimerin (chimaerin) 2	0.16	0.12	0.29	0.14	0.08	0.22	0.11	0.01	0.02	0.03	**0.58**	0.08	1.1	1.4	1.0	0.8	0.008	0.06	0.05
Podxl	Podocalyxin-like	0.12	0.14	0.01	0.13	0.20	0.07	0.10	0.19	**0.57**	**0.60**	0.02	**0.68**	1.2	0.7	0.9	1.6	0.002	0.02	0.02
Eif4e3	Eukaryotic translation initiation factor 4E member 3	0.12	0.14	0.01	0.14	0.20	0.07	0.10	0.18	**0.57**	**0.59**	0.02	**0.68**	0.9	1.2	1.0	0.8	0.002	0.02	0.02
Tmem140	Transmembrane protein 140	0.10	0.04	0.31	0.03	0.01	0.11	0.12	0.06	0.03	0.04	0.43	0.08	0.7	0.5	1.4	2.0	0	0	0.00001
Kel	Kel Kell blood group	0.10	0.10	0.01	0.12	0.17	0.04	0.02	0.02	0.36	0.36	0.03	0.43	1.1	0.8	1.2	1.6	0	0	0.00001
Abcg2	ATP-binding cassette, sub-family G (WHITE), member 2	0.09	0.03	0.29	0.02	0.01	0.09	0.11	0.05	0.04	0.04	0.40	0.09	0.9	0.8	1.1	1.2	0.005	0.06	0.05
Tia1	Cytotoxic granule-associated RNA binding protein 1	0.06	0.06	0.00	0.11	0.07	0.08	0.00	0.15	0.03	0.05	0.17	0.08	1.0	1.2	1.3	1.1	0.001	0.02	0.02
Serbp1	Serpine1 mRNA binding protein 1	0.05	0.05	0.02	0.05	0.09	0.01	0.01	0.04	0.36	0.37	0.08	0.46	1.0	0.9	1.0	1.2	0.0007	0.01	0.01
Hoxa5	Homeo box A5	0.05	0.00	0.17	0.00	0.01	0.01	0.05	0.05	0.08	0.08	0.18	0.12	1.2	1.6	0.7	0.5	0	0	0.00004
Foxp1	Forkhead box P1	0.05	0.02	0.08	0.03	0.05	0.00	0.01	0.00	0.18	0.18	0.11	0.23	1.0	1.2	0.9	0.7	0.01	0.06	0.05
Bola3	BolA homolog 3	0.02	0.00	0.09	0.00	0.00	0.03	0.00	0.02	0.19	0.22	0.28	0.30	1.0	0.9	1.0	1.1	0.009	0.08	0.07
Predicted genes	Predicted genes																			
Ppp4r2	Protein phosphatase 4, regulatory subunit 2	**0.74**	**0.76**	**0.80**	**0.68**	**0.72**	**0.74**	**0.94**	**0.97**	**0.85**	**0.86**	0.49	**0.80**	1.1	1.0	0.9	1.0	0.004	0.03	0.03
Mpp6	Membrane protein, palmitoylated 6 (MAGUK p55 subfamily member 6)	**0.73**	**0.76**	**0.76**	**0.68**	**0.72**	**0.72**	**0.92**	**0.96**	**0.89**	**0.89**	0.44	**0.84**	0.7	1.0	1.3	1.0	0.00008	0.001	0.002
Baiap1	BAI1-associated protein 1	0.43	0.45	0.49	0.37	0.43	0.39	0.67	0.91	**0.80**	**0.83**	0.17	**0.83**	0.7	1.1	1.4	0.9	0.001	0.05	0.04
Cct7	Chaperonin subunit 7	0.39	0.45	0.14	0.45	**0.53**	0.32	0.33	0.34	**0.82**	**0.82**	0.05	**0.86**	1.1	0.9	1.0	1.2	0.00007	0.001	0.002
Chchd6	Coiled-coil-helix-coiled-coil-helix domain containing 6	0.38	0.44	0.08	0.47	**0.53**	0.32	0.23	0.17	**0.67**	**0.65**	0.05	**0.68**	1.2	0.9	1.1	1.4	0.004	0.03	0.03
MGC94941	Similar to Mkrn1 protein	0.31	0.35	0.04	0.39	0.44	0.24	0.14	0.08	**0.54**	**0.52**	0.02	**0.55**	1.2	0.9	1.2	1.7	0	0	0.0002
Slc35b4	Predicted solute carrier family 35, member B4	0.19	0.12	0.50	0.09	0.06	0.21	0.29	0.24	0.00	0.00	0.50	0.00	1.2	1.2	0.9	0.8	0.0006	0.007	0.008
RGD1563542	Similar to Kell protein	0.14	0.15	0.00	0.18	0.23	0.08	0.03	0.01	0.36	0.35	0.01	0.40	1.1	0.8	1.3	1.8	0.00003	0.0006	0.0007
Nt5c3	Predicted 5′-nucleotidase, cytosolic III	0.14	0.07	0.41	0.05	0.03	0.14	0.21	0.16	0.00	0.00	0.43	0.02	0.7	0.6	1.5	1.8	0	0	0
RGD1311080	Similar to RIKEN cDNA A930038C07	0.12	0.12	0.01	0.16	0.19	0.06	0.01	0.00	0.22	0.21	0.00	0.24	1.0	1.2	0.8	0.7	0.00008	0.001	0.002
Ube2h	ubiquitin-conjugating enzyme E2H	0.09	0.11	0.00	0.11	0.16	0.04	0.06	0.13	0.50	**0.52**	0.03	**0.61**	1.1	0.8	1.0	1.3	0.008	0.07	0.06
RGD1306936	Similar to chromosome 7 open reading frame 30	0.06	0.00	0.25	0.00	0.00	0.03	0.12	0.19	0.01	0.00	0.17	0.01	1.1	1.2	0.8	0.8	0.003	0.03	0.02

a Genes (*r*^2^ > 0.50) are indicated in bold face.

b P allele is high bone mass expressing genotype.

c FDR-Chr 4: False discovery rate from the analysis of 460 probe sets in the chromosome 4 QTL region.

d FDR-Chr all: False discovery rate from the analysis of all probe sets in all chromosomes.

#### Pathway analysis

**Fig. 1:**
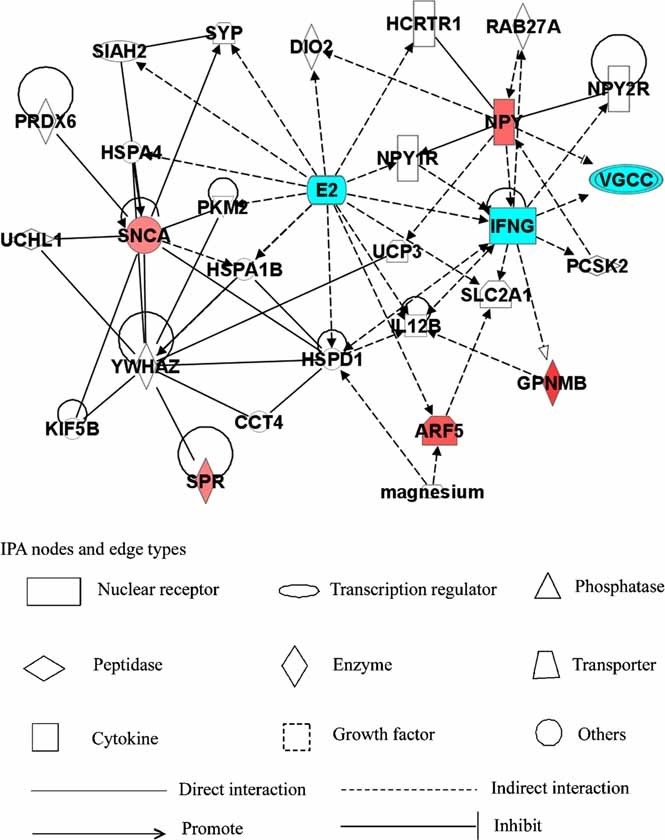
Of nine candidate genes that were highly correlated (*r*^2^ > 0.5) with average BMC (Table 3), a network of five eligible genes (*Snca*, *Spr*, *Npy*, *Arf5*, and *Gpnmb*) was shown in Ingenuity pathway analysis (IPA). Well-known pathways related to bone metabolism are highlighted in green .

The nine candidate genes that were highly correlated with bone phenotypes at multiple skeletal sites were mapped to pathways using Ingenuity. Among the nine candidate genes that were highly correlated with bone phenotypes at multiple skeletal sites, six genes (*Snca*, *Spr*, *Npy*, *Arf5*, *Gpnmb*, and *Fkbp14*) were identified by Ingenuity pathway for network analysis. *Fkbp14* was not linked to the molecules within the same pathways. The remaining five genes that were eligible for network analysis were directly or indirectly connected to pathways related to *β-estradiol* (*E2*), *interferon-γ* (*IFNG*), and *voltage-gated calcium channel* (*VGCC*) (Fig. 1).

#### Real-time quantitative PCR (qPCR) analyses

qPCR analyses of the top three candidate genes in Table 3 using RNA from femurs confirmed the strong correlation between gene expression and bone mass and strength phenotypes (Table 4). The correlation coefficients for *α-synuclein* (*Snca*), *sepiapterin reductase* (*Spr*), and *neuropeptide Y* (*Npy*) between microarray and qPCR analyses were 0.87, 0.87, and 0.91, respectively, indicating good agreement between the two methods. The whole-femur RNA comes from a variety of different types of cells, and thus we cannot be sure that the gene-expression changes originated from bone cells. To address this problem, we cultured primary osteoblasts from rat calvaria and performed qPCR analysis of gene expression for the three candidate genes. We observed similar correlations between gene expression and bone phenotypes, suggesting that osteoblastic cells are the main regulators for bone mass and strength in these rats.

**Table 4:** R-square values and fold-change for top 3 candidate genes in femur and primary osteoblast by q-PCR in NP, NP.P, P.NP and P rats^a^^b^

		Skeletal phenotypes												Fold change			
			
Gene symbol	Gene name	Average BMC	Whole body BMC/BW	Cranial BMC/BW	Femur BMC/BW	L1-6 BMC/BW	Whole body aBMD	Cranial aBMD	Femur Midshaft Total vBMD	Distal Femur Total vBMD	L5 Total vBMD	Femur Ultimate Force	Femur Neck Ultimate Force	NP vs P	NP vs NP.P	P vs P.NP	NP.P vs P.NP
Femur																	
Snca	Synuclein, alpha	**0.93**	**0.98**	**0.76**	**0.99**	**0.97**	**0.98**	**0.80**	0.48	**0.62**	**0.57**	**0.81**	0.48	2.0	1.4	0.8	1.2
Spr	Sepiapterin reductase	**0.88**	**0.94**	**0.63**	**0.98**	**0.96**	**0.92**	**0.69**	0.37	**0.61**	**0.56**	**0.71**	0.48	2.3	1.4	0.9	1.5
Npy	Neuropeptide Y	**0.90**	**0.96**	**0.65**	**0.99**	**0.98**	**0.93**	**0.74**	0.44	**0.69**	**0.64**	**0.68**	**0.56**	2.1	1.3	0.9	1.4
Primary osteoblast																	
Snca	Synuclein, alpha	**0.71**	**0.76**	**0.61**	**0.70**	**0.77**	**0.68**	**0.82**	**0.84**	**0.98**	**0.98**	0.34	**0.96**	2.2	1.0	0.6	1.3
Spr	Sepiapterin reductase	**0.68**	**0.64**	**0.96**	**0.57**	**0.54**	**0.72**	**0.86**	**0.75**	0.38	0.37	**0.80**	0.29	0.5	0.6	1.6	1.3
Npy	Neuropeptide Y	**0.54**	**0.59**	0.44	**0.53**	**0.61**	**0.50**	**0.68**	**0.80**	**0.96**	**0.98**	0.18	**0.98**	1.3	0.9	0.8	1.1

a Genes correlated *r*^2^ > 0.50 are indicated in bold face

^a,b^P allele is high bone mass expressing genotype

#### Cis- and trans-regulated genes

The preceding analyses focused on *cis*-regulated genes, that is, genes within the QTL region that were differentially expressed (Table 3). We also evaluated *trans*-regulated genes by comparing differentially expressed genes between inbred (NP and P) and their corresponding congenic (NP.P and P.NP) rats. A total of seven genes outside the QTL region (*trans*-) were differentially expressed between NP and NP.P rats at FDRs of less than 10% (Table 5). No *trans*-regulated genes were identified in the comparison of P with P.NP rats.

**Table 5:** Trans-regulated genes between NP and NP.P rats with a false discovery rate (FDR) less than 10%^a^

Gene symbol	Gene name	Fold change	*p*-value	FDR	Rat genome location
Ptgfrn	Prostaglandin F2 receptor negative regulator	1.47	0.00001	0.04	2q34
Ddx58	DEAD (Asp-Glu-Ala-Asp) box polypeptide 58	−1.31	0.00001	0.03	5q22
Otub2	OTU domain, ubiquitin aldehyde binding 2	1.27	0.00001	0.04	6q32
DnaJ	Hsp40 homolog, subfamily B, member 2	1.19	0.00001	0.03	9q33
Bnip3l	BCL2/adenovirus E1B 19 kDa-interacting protein 3-like	1.11	0.00005	0.08	15p12
F13a	Coagulation factor XIIIa	−1.36	0.00005	0.08	17p12
RT1-Db1	RT1 class II, locus Db1	−1.52	0.00002	0.04	20p12

a P allele is high bone mass expressing genotype.

## Discussion

Our results demonstrated that transfer of the NP chromosome 4 QTL (q22–q34) onto the P background (P.NP) significantly increased body weight but decreased BMD at several skeletal sites. Conversely, transfer of the P chromosome 4 QTL onto the NP background (NP.P) significantly decreased body weight but increased bone density at the same skeletal sites, indicating that the chromosome 4 QTL harbors a gene or genes that affect bone mass and structure. In addition, we identified several candidate genes, including some novel genes located within the 4q22–q34 QTL region, that are differentially expressed and strongly correlated with skeletal phenotypes in congenic P and NP rats. We also confirmed the top three candidate genes (*Snca*, *Spr*, and *Npy*) by qPCR.

Recently, there has been considerable interest in a link between brain and bone. Several studies have shown that hormones produced in the brain regulate bone mass through neuroendocrine pathways.(23–25) Interestingly, two of the top candidate genes (*Snca* and *Npy)* identified in this study were found to be differentially expressed not only in bone cells but also in brain tissue,(19,30–32) indicating a possible link between neuronal signaling and skeletal regulation of bone mass in these rats. *Snca* has been shown to be associated with alcohol preference in rats(19) and with craving in alcoholics,(20,21) as well as with upregulation matrix mineralization in the human osteosarcoma cell line.(22) Interestingly, *Npy* affects body weight, alcohol preference, anxiety, and bone mass and strength. The effect of *Npy* on BW regulation was demonstrated by several studies in mice,(26,27) with high expression of *Npy* resulting in increased BW. A null mutation in *Npy* increased alcohol preference in mice,(28,29) and lower levels of *Npy* expression in discrete brain regions in P rats was associated with higher alcohol consumption.(30,31) Furthermore, there is an association between the decreased level of neuropeptide Y and increased anxiety in P rats,(33) and transfer of the P chromosome 4 QTL onto the NP background caused increased anxiety in NP.P rats compared with NP rats (unpublished data). Several studies have demonstrated that neuropeptide Y regulates bone mass by an apparent neuronal pathway.(34,35) Mice with impaired *Npy* signaling had higher cortical and trabecular bone mass at different skeletal sites.(36–38) In humans, a common polymorphism in leucine 7 to proline 7 in prepro-*Npy* gene (Leu7Pro7) was found to be associated with alcohol dependence(39,40) and higher femoral neck BMD in postmenopausal women.(41) In addition, neuropeptide Y–receptor genes are associated with alcohol dependence and withdrawal phenotypes.(42) All these studies suggest that neuropeptide Y falls within a common genetic pathway affecting bone mass, body weight, anxiety, and alcohol preference.

It is well established that increased physical activity is associated with decreased BW(43) and higher bone mass in humans.(44) However, the effect of activity is mostly restricted to weight-bearing skeletal sites,(45) and non-weight-bearing sites such as the cranium are not strongly affected. Consequently, the variation in bone mass at the cranium is more likely to be related to genetic factors rather than the biomechanical effects of activity. We found that P rats were more active than NP rats, and transfer of the P chromosome 4 onto the NP background made NP.P rats more active than NP rats. In addition, we found that P rats had higher BMD than NP rats at several different skeletal sites, and transfer of the P chromosome 4 onto the NP background decreased bone mass in NP.P rats at the same sites, suggesting that activity level might influence the BMD in P, NP and congenic rats. The correlation coefficients between activity and whole-body aBMD, L_1_–L_6_ aBMD, femur midshaft total vBMD, distal femur total vBMD, and L_5_ total vBMD were 0.97, 0.88, 0.61, 0.68, and 0.65, respectively. These results suggest that the biomechanical effects of activity play some role in the regulation of bone mass in these rats. We also looked at bone density in the cranium to evaluate genetic influences that are independent of weight bearing. We detected significantly higher BMD values in the crania of P rats compared with NP rats, and transfer of the QTL region in both directions (NP.P and P.NP) was associated with significantly increased or decreased bone mass in the cranium compared with the background strains (NP and P). These findings suggest a direct genetic effect on bone density, independent of the biomechanical effects caused by alterations in activity levels.

Since the BWs of P, NP, and the congenic rats were significantly different and negatively correlated with BMD at different skeletal sites, we normalized some of the bone-mass phenotypes by BW to allow comparisons among the strains. The normalized phenotypes included several BMC measurements (bone mass) and polar moment of inertia (bone size). While it is quite reasonable to normalize measures of bone mass or size by BW, we recognize that such normalization could bias our results by creating composite phenotypes that do not represent true bone traits. Therefore, we were careful to compare the normalized BMC measurements with the vBMD measures taken from pQCT. We found that the normalized values of BMC correlated well with the vBMD values across different skeletal sites, suggesting that the normalization method did not distort these skeletal phenotypes.

In this study we used young (4-week-old) rats rather than adult rats (26 weeks old) in the gene-expression study because gene expression is substantially suppressed in the mature skeletons in adult rats. We targeted a rapid skeletal growth phase so that the gene expression should reflect the accrual of bone toward peak bone mass obtained at 26 weeks and for which we detected QTLs. Among nine candidate genes that were highly correlated with bone phenotypes at multiple skeletal sites, a genetic network of five eligible genes (*Snca*, *Spr*, *Npy*, *Arf5*, and *Gpnmb)* was associated with direct or indirect pathways controlling cell morphology, cell proliferation, integrin signaling, cellular organization, receptor signaling, molecular transport, and organ development (Fig. 1). Genes in the canonical pathways (network generated in the IPA and known pathways that were associated with metabolism or signaling) were related to serotonin and dopamine receptor signaling, protein kinase C inhibitor and integrin-mediated signaling, folate biosynthesis, and arginine and proline metabolism. When these genes were categorized based on location or cellular components *Snca*, *Spr*, and *Arf5* were located in cytoplasm, whereas *Npy* and *Gpnmb* were located in the plasma membrane. Interestingly, we detected several pathways directly or indirectly related to the candidate genes we obtained from this study with the genes already reported to be related to bone metabolism (highlighted in green). Among them, the pathway related to *β-estradiol* (*E2*) has been shown extensively to regulate bone density and turnover.(46–48) *Interferon-γ* inhibits osteoclastogenesis,(49) and *voltage-gated calcium channel* pathway is essential for chondrocyte proliferation and differentiation(50) and mediates mechanical load–induced bone formation.(51) Gremlin 1 interacts with tyrosine 3-monooxygenase/tryptophan 5-monooxygenase activation protein (YWHAZ) and acts as an antagonist of the bone morphogenetic protein (BMP) pathway.(52) Identifying the molecular mechanism by which *Npy* regulates estrogen and calcium channel pathways and *Snca* and *Spr* modulate the BMP pathway thus will be valuable for understanding the skeletal homeostasis controlled by these genes. Also, further investigation of bone phenotypes in knockout or transgenic animal models involving these genes will provide important insight for their role in bone-mass regulation. Additionally, gene silencing using siRNA or overexpression of these genes in cell culture systems can be undertaken for functional characterization of these genes.

In this study we prioritized candidate genes by analyzing the microarray-based expression of genes underlying the QTLs at rat 4q22–q34 and correlating them with multiple skeletal phenotypes. In another study using the same method, we investigated the genomic expression for skeletal traits at femoral neck in F344 and LEW strains.(53) Interestingly, we detected two genes, *Spr* and *glycoprotein (transmembrane) nmb* (*Gpnmb)* that were significantly (*r*(2) > 0.50) correlated with skeletal phenotypes in both P/NP congenics and F344/LEW rats. Ranking of the microarray-based candidate genes is usually performed by analyzing the magnitude of expression differences (fold differences) between different strains. However, with complex traits such as osteoporosis, even subtle changes in gene expression could be important, and therefore, a larger expression difference might not necessarily identify the best candidate genes. We believe that the correlation between gene expression and physical traits provides stronger evidence for the association between a gene and a trait.

Most of the genes we identified in this study were *cis*-acting, but we also found some novel *trans*-regulated genes between NP and NP.P rats. However, besides these *trans*-regulated genes, some other potential *trans*-acting genes might be influencing bone phenotypes in these rat strains because the bone parameters were not consistently different between NP and NP.P and between P and P.NP rats for both DXA and pQCT measurements (Table 2). *Cis*-acting polymorphisms are located at or near the gene that exhibits altered expression levels. *Trans*-acting regulation involves polymorphisms within the QTL region that affect gene expression outside the QTL. *Trans*-acting genes can provide unique information about the gene networks influencing complex phenotypes.(54) The role of these *trans*-regulated genes in bone metabolism is yet to be discovered.

In conclusion, using P and NP congenic rats, we demonstrated that several candidate genes, including some novel genes located within rat 4q22–q34, are differentially expressed and strongly correlated with bone density. Among these genes, *Npy* is a likely common genetic modulator for bone density, body weight, activity, and alcohol preference. However, our approach has several limitations. Identification of candidate genes by correlating differential gene expression with the various skeletal phenotypes simply provides us with a list of potential candidate genes for further prioritization. Moreover, gene-expression study based on microarray analysis explains only transcriptional regulation of genes and does not capture the effects of alternative gene splicing, polymorphism in the coding region affecting protein structure and function, or posttranslational modification of proteins. Also, whether the same candidate genes regulate skeletal phenotypes in female rats remains to be determined because we studied only male rats in this study. Further studies involving the molecular mechanism by which the genes identified in this study regulating bone mass thus are necessary for the development of drugs to prevent and treat osteoporosis.
